# Bioinformatics and Experimental Analysis of the Prognostic and Predictive Value of the CHPF Gene on Breast Cancer

**DOI:** 10.3389/fonc.2022.856712

**Published:** 2022-03-15

**Authors:** Wan-Wan Li, Bin Liu, Shu-Qing Dong, Shi-Qing He, Yu-Ying Liu, Si-Yu Wei, Jing-Yi Mou, Jia-Xin Zhang, Zhao Liu

**Affiliations:** ^1^ Department of General Surgery, The Affiliated Hospital of Xuzhou Medical University, Xuzhou, China; ^2^ Institute of Digestive Diseases, Xuzhou Medical University, Xuzhou, China

**Keywords:** bioinformation, breast cancer, CHPF, prognosis, immune, DNA methylation

## Abstract

**Background:**

Recent studies in the United States have shown that breast cancer accounts for 30% of all new cancer diagnoses in women and has become the leading cause of cancer deaths in women worldwide. Chondroitin Polymerizing Factor (CHPF), is an enzyme involved in chondroitin sulfate (CS) elongation and a novel key molecule in the poor prognosis of many cancers. However, its role in the development and progression of breast cancer remains unclear.

**Methods:**

The transcript expression of CHPF in the Cancer Genome Atlas-Breast Cancer (TCGA-BRCA), Gene Expression Omnibus (GEO) database was analyzed separately using the limma package of R software, and the relationship between CHPF transcriptional expression and CHPF DNA methylation was investigated in TCGA-BRCA. Kaplan-Meier curves were plotted using the Survival package to further assess the prognostic impact of CHPF DNA methylation/expression. The association between CHPF transcript expression/DNA methylation and cancer immune infiltration and immune markers was investigated using the TIMER and TISIDB databases. We also performed gene ontology (GO) annotation and Kyoto Encyclopedia of Genes and Genomes (KEGG) pathway analysis with the clusterProfiler package. Western blotting and RT-PCR were used to verify the protein level and mRNA level of CHPF in breast tissue and cell lines, respectively. Small interfering plasmids and lentiviral plasmids were constructed for transient and stable transfection of breast cancer cell lines MCF-7 and SUM1315, respectively, followed by proliferation-related functional assays, such as CCK8, EDU, clone formation assays; migration and invasion-related functional assays, such as wound healing assay and transwell assays. We also conducted a preliminary study of the mechanism.

**Results:**

We observed that CHPF was significantly upregulated in breast cancer tissues and correlated with poor prognosis. CHPF gene transcriptional expression and methylation are associated with immune infiltration immune markers. CHPF promotes proliferation, migration, invasion of the breast cancer cell lines MCF-7 and SUM1315, and is significantly enriched in pathways associated with the ECM-receptor interaction and PI3K-AKT pathway.

**Conclusion:**

CHPF transcriptional expression and DNA methylation correlate with immune infiltration and immune markers. Upregulation of CHPF in breast cancer promotes malignant behavior of cancer cells and is associated with poorer survival in breast cancer, possibly through ECM-receptor interactions and the PI3K-AKT pathway.

## Introduction

Breast cancer has become the second most common cause of cancer death in women worldwide (1). According to previous literature, there is no clear cause of breast cancer to achieve precise cause-specific treatment; some patients are still at advanced stages upon detection due to the limitations of early diagnosis techniques and popularity; some types of breast cancer progress rapidly and have limited treatment options.

The emergence of prognostic predictors is expected to improve the prognosis of breast cancer patients. Previous clinical applications mostly relied on tumor size, lymph node status, and tumor grading, which were later found not to enable personalized treatment. Therefore, the search for new markers that can achieve a prognostic role has gradually tended to continue, from the RNA level to the protein level. However, none of these studies has achieved a revolutionary breakthrough, and there is still an urgent need for more emerging indicators.

Efforts have been made by experts from various disciplines to improve the prognosis of breast cancer patients. For example, the introduction of new diagnostic techniques (2, 3); the introduction of genomic and metabolomic studies thus refining the type of breast cancer pathology (4–6); the exploration of molecular markers (7–10) and the development of targeted therapeutic modalities (11–16). However, there is a lack of more studies about the emerging molecular marker, CHPF, in cancer.

Chondroitin Sulfate(CS), is a type of sulfated glycosaminoglycans (GAGs) (17, 18) and is involved in the biosynthesis of the skeleton (19). Multiple studies find CS involvement in tumor progression and metastasis (20–23). CHPF has beta-1,3-glucuronic acid and beta-1,4-N-acetylgalactosamine transferase activity and is involved in CS chain elongation (24–26). CHPF is located in the 2q35-q36 region of human chromosomes, spanning four exon regions, and plays an important role in cellular function (27).The latest study reported that, CHPF may act as both an oncogene and a cancer-promoting factor in a variety of tumors. It is upregulated and its high expression was positively correlated with poor prognosis in breast cancer (28, 29), lung cancer (30–32), malignant melanoma (33), cholangiocarcinoma (34). However, studies in hepatocellular carcinoma are contradictory (35, 36). Currently, there is no clear mechanism of action of CHPF in cancer. In addition, there are few studies on this gene in breast cancer, and there is a lack of additional evidence to confirm its important role in breast cancer.

In this paper, we investigated the role of CHPF in breast cancer prognosis prediction and proposed a combined bioinformatics and basic experimental approach. The present study presents and explores for the first time the relevance of CHPF as well as methylation to immunity.

## Methods and Materials

### UCSC Xena

UCSC Xena (http://xena.ucsc.edu/) comprises a cancer genomics data analysis platform containing integrated data from various TCGA tumors. Obtaining breast cancer expression data, survival data files, and pan-cancer data from this site.

### CHPF DNA Methylation and Cancer Immune Infiltration Analysis

The relationship between CHPF DNA methylation and CHPF transcript expression was investigated in TCGA-BRCA. KM survival analysis was performed using Survival package to evaluate the potential impact of CHPF DNA methylation/expression on clinical outcomes. Analysis of the association between CHPF transcript expression/DNA methylation and cancer immune infiltration using the GSCA database.

### Source of Human BRCA With Adjacent Non-Tumor Samples

All breast tissue samples were obtained from the Department of Pathology, Affiliated Hospital of Xuzhou Medical University. The patients’ clinicopathological data were obtained from the hospital medical record system and informed consent was obtained from patients for all human samples. The specimens and data used for the study were approved by the hospital ethics committee.

### Cell Lines and Cell Culture

The breast cell lines MCF-7, MCF-10A, MDA-MB-231, SUM1315, ZR-75-1, T47D were purchased from the Shanghai Institute of Biochemistry and Cell Biology, Chinese Academy of Sciences (Shanghai, China). MCF-7, MDA-MB-231, SUM1315, ZR-75-1 and T47D cells were cultured in DMEM medium (with 10% fetal bovine serum and 1% Penicillin-streptomycin solution), whereas MCF-10A cells were cultured in DMEM/F12 medium supplemented with 10% fetal bovine serum and were incubated in a 37°C humidified incubator with 5% CO2.

### RNA Extraction and RT-qPCR

Total RNA was extracted using the TRIzol reagent (Invitrogen) according to instructions. After determining the RNA concentration, the reverse transcription reaction was performed with HiScript II Q RT SuperMix for qPCR (Vazyme Biotech, Nanjing, China). The RNA expression was determined using SYBR Green (Vazyme Biotech, Nanjing, China) reagents. The machine was operated on a Bio-Rad QX100 Droplet Digital PCR system (USA) and the relative RNA amounts were calculated and normalized to GAPDH using the 2^-ΔΔCt^ method. All premiers were obtained from GENERAY Biotechnology (Shanghai, China) and are summarized in **Additional File 1: Table S1**.

### Western Blotting

Total proteins were extracted from tissues or cells using pre-cooled RIPA buffer (Beyotime, Shanghai, China) containing protease inhibitors (Thermo Scientific, USA). Protein quantification was performed with a dicinchoninic acid protein assay kit (Thermo Scientific, USA). Equal amounts of protein samples were separated by the 4-12% SDS-PAGE (GenScript, Nanjing, China) and then transferred to 0.45μm PVDF membranes (Millipore, USA). After blocking with TBST containing 5% skim milk for 2h, the membranes were incubated with the corresponding primary antibodies overnight at 4°C. TBST was washed 3 times and incubated with HRP-coupled secondary antibodies at room temperature for 1h. Immunoblots were detected by an imaging system (Bio-Rad, USA) using an enhanced chemiluminescence detection kit (Servicebio, Wuhan, China). GAPDH was selected as a loading control. Primary antibodies specific for CHPF (ab224495) were purchased from Abcam. Anti-GADPH (#51332), anti-E-cadherin (#2195), anti-N-cadherin (#13116), anti-Vimentin (#5741), anti-Snail (#3879), anti-PI3K(#4249), anti-AKT(#4691), and anti-p-AKT(#S473) were purchased from Cell Signaling Technology. Anti-phospho-PI3K(Tyr485) (sc-130211) was purchased from Santa Cruz Biotechnology (USA). The secondary goat anti-rabbit or goat anti-mouse (Abcam, USA).

### siRNA Transfections

CHPF (siLCHPF) specific small interfering RNA (siRNA) and non-specific control siRNA (siCtrl) were purchased from GenePharma (Shanghai, China) and transfected with siLentFect Lipid Reagent (Bio-Rad Laboratories, Inc.) based on the manufacturer’s instructions when BRCA cells grew to 20~50% confluence. Four to six hours after transfection, the medium containing the transfection reagent was substituted with a medium containing 10% fetal bovine serum. The siRNA sequences are listed in **Supplementary Table S2**.

### Stable Cell Line Generation

CHPF short hairpin RNA (shRNA) and interference control lentivirus were purchased from GenePharma. Cells were spread in 24-well plates at 1×10^5/well. The next day, 2 ml of fresh medium containing polybrene 6-8 ug/ml was added to replace the original medium, followed by the addition of an appropriate amount of virus suspension and incubation at 37°C. After 4-6 h, the medium was changed. Continue incubation for 24-48 h and then screen with 2 ng/ml puromycin for 2 weeks, changing the medium every 3 days. Stably transfected cell lines were screened. The infected and screened cells were passaged and continued to be cultured with the addition of puromycin for maintenance screening, and after 3 generations of continuous culture and passaging, the cells were lyophilized. The shRNA sequences are listed in **Supplementary Table S3**.

### Cell Proliferation Assay and Colony Formation Assay

To assess the proliferative capacity of cells, Cell Counting Kit-8 (CCK-8, Dojindo Laboratories, Kumamoto, Japan) was used. Cells were inoculated into 96-well plates at 2000 cells per well. Six replicated wells were set up for each group. Then,10μl CCK8 solution was added to the wells and the samples were incubated for 2 h at 37°C. The absorbance of the samples at 450 nm was measured for five consecutive days. We performed three independent experiments and presented the results as mean ± SD. For colony formation assays, 800 cells/well were inoculated in 60 mm plates and cultured in a medium containing 10% FBS for 14 days. The culture medium was discarded, methyl-fixed for 20 min, stained with crystal violet for 20 min, gently rinsed in running water, dried, and photographed for counting.

### EDU Assays

EDU (5-Ethynyl-2’-deoxyuridine) assays were performed using the EDU assay kit (RiboBio, Guangzhou, China) and according to the manufacturer’s instructions. Logarithmic growth phase cells were taken and cultured at 4 × 10^3^ cells/well and inoculated in 96-well plates. After 20h of incubation, cells were treated with 50μmol/L Edu medium and incubated at 37°C for 2 h. Cells were washed with PBS, fixed with 4% paraformaldehyde for 30 min, and incubated with 50 ul of 2 mg/mL glycine for 5 min. The cells were then incubated with 0.5% Triton X-100 permeate for 10 min and 100μL of 1× Apollo^®^ staining reaction solution for 30 min at room temperature, protected from light, followed by permeabilization. Finally, 100μL of Hoechst 33342 (5μg/mL) was used for staining for 30 min and observed and captured with a fluorescence microscope (IX71; Olympus, Tokyo, Japan).

### Wound Healing Assay

The cells were cultured to logarithmic phase and then inoculated in 6-well plates according to 1×105 cells per well and incubated in an incubator at 37°C for 24 h. After the cells were spread all over, a 20.0 μL pipette tip was used to scratch vertically on the horizontal line, and the medium without fetal bovine serum was added after washing with PBS, and the position and width of the scratch were recorded under a 200× microscope at 0 h. The cells were further incubated in the incubator for 24 h and then recorded under 200× microscope. After incubation for 24 h, the cells were photographed and recorded under 200× microscope to detect the migration distance.

### Transwell Assay

Transwell (BD bioscience, SanJose, CA)assay was used to assess cell invasiveness. The matrigel was diluted in proportion (serum-free medium: matrigel = 9:1) one day before performing the invasion assay and the diluted matrigel was subsequently added to the upper chamber (40ul/well) and placed in a 37°C incubator overnight. Cells were counted and 40,000 cells/well were selected for invasion assay, and added to 200ul with serum-free medium. At the same time, 800 ul of the serum-containing medium was added to the lower chamber. Placing 24-well plates in a 37°C incubator for 48 hours. When the time comes, fixation, staining, swabbing, and photo-counting are performed. Five randomly selected regions were counted for the number of invading cells.

### Bioinformatical and Statistical Analysis

All statistical analyses of bioinformatics were performed with Rstudio software (version 1.4.1717; http://www.rstudio.com/products/rstudio). First, differential expression analysis was performed using the limma package to explore whether CHPF is differentially expressed in breast cancer patients and normal cases. To explore the correlation between CHPF transcriptional expression/DNA methylation and the prognosis of breast cancer patients, Kaplan-Meier survival analysis was performed in this study using the Survival and Survminer software packages and matched by the log-rank test. In addition, we further analyzed the univariate Cox regression analysis between multivariate and survival. In order to explore the possible mechanism of action of CHPF in breast cancer, we performed GO,KEGG, and GSEA analysis based on TCGA data. All underlying experimental statistical analyses were performed by the SPSS 23.0 statistical package (SPSS, Inc., Chicago, IL). T-tests were used to evaluate differences between control and knock-down groups. Differences were deemed significant when P < 0.05.

## Results

### CHPF Is Highly Expressed in Breast Cancer and Is Associated With Poor Prognosis

**Figure 1:**
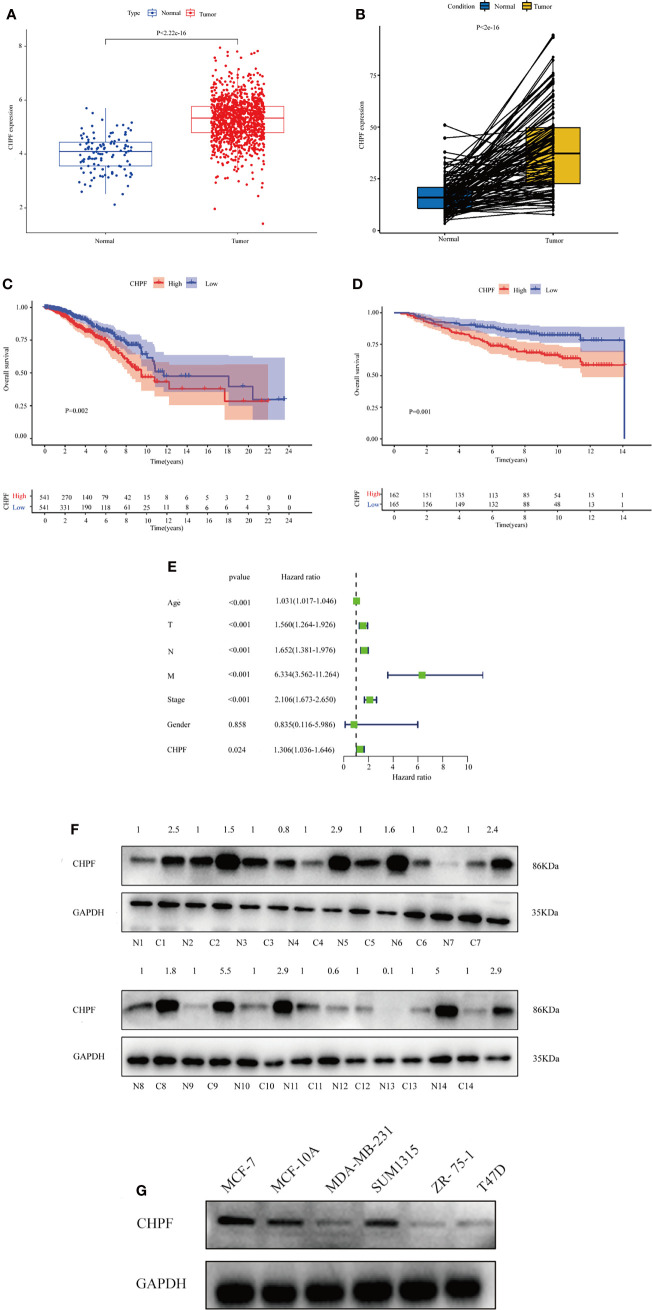
Expression of CHPF is significantly higher in breast cancer and negatively correlated with overall survival. **(A)** ATCGA data analysis shows high expression of CHPF gene in breast cancer tissues. **(B)** Expression levels of CHPF genes in paired breast samples. **(C)** The results of KM survival analysis based on TCGA-BRCA data (p = 0.002, log-rank test). **(D)** The results of KM survival analysis based on breast cancer GEO dataset GSE20685 (P = 0.001, log-rank test). **(E)** Univariate COX regression analysis shows CHPF gene expression as a poor prognostic factor. **(F)** CHPF protein expression was detected by western blotting in fourteen paired LUAD tissue. **(G)** Western blotting detected the protein levels of CHPF in six breast cell lines.

After preprocessing the data from 33 tumors obtained from UCSC Xena, differential expression analysis was performed using the limma package to compare CHPF expression in 33 tumor samples as well as the corresponding normal samples (in this case, only tumors with the number of normal samples >= 5 were selected).Significant differences in CHPF gene expression were found in BLCA, BRCA, CHOL, COAD, ESCA, GBM, HNSC, KICH, KIRC, KIRP, LIHC, LUAD, LUSC, PRAD, READ, STAD, and UCEC, and the gene expression was significantly upregulated in breast cancer (**Figure 1A**). Because of the unequal number of TCGA-BRCA tumor samples and normal samples, a paired analysis was selected, which showed a significant increase in CHPF gene expression levels in breast tumor samples (**Figure 1B**). Subsequent KM survival analysis was performed and the results demonstrated a poorer prognosis in the high CHPF expression group (**Figure 1C**), in addition, we selected the GEO dataset GSE20685 for further validation and obtained the same results as TCGA (**Figure 1D**). When we integrated both clinicopathological factors and CHPF gene expression in the univariate Coxregression analysis variables, we could see that CHPF gene expression was related to prognosis and function as a risk factor (**Figure 1E**). Here, we analyzed the expression of the CHPF gene in 14 pairs of tumors and normal tissues and found that the CHPF gene was significantly increased in breast cancer tissues (**Figure 1F**). In addition, we examined CHPF protein levels in six breast cell lines by western blotting. CHPF was extensively abundant in breast cancer cell lines MCF-7 as well as SUM1315(**Figure 1G**), and so these two cell lines were selected for follow-up studies.

### CHPF DNA Methylation Was Negatively Correlated With CHPF Transcript Expression, Both of Which Were Associated With Immune Infiltrates

**Figure 2:**
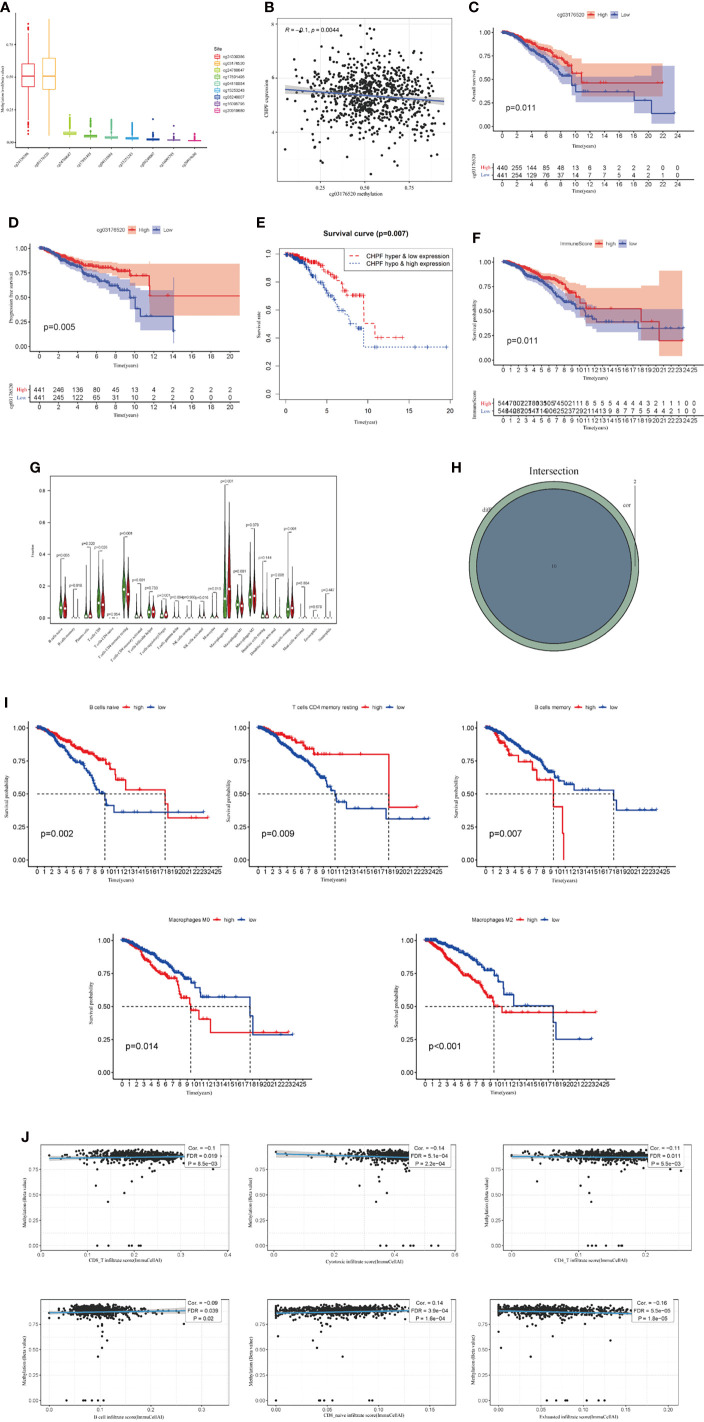
CHPF gene is associated with DNA methylation, and immune infiltration. **(A)** The extent of methylation at different loci in the CHPF gene. **(B)** CHPF gene expression is negatively correlated with methylation sites cg03176520. **(C)** Overall survival is higher in breast cancer patients with high cg03176520 methylation. **(D)** PFS is higher in breast cancer patients with high cg03176520 methylation. **(E)** Breast cancer patients with both high CHPF gene expression levels and hypomethylation have a poorer prognosis. **(F)** Scoring of immune cells in the tumor microenvironment in TCGA-BRCA. **(G)** Differential expression of immune cells in high and low subgroups of CHPF genes. **(H)** Results of correlation analysis between CHPF gene and immune cells. **(I)** Positive results of KM survival analysis of immune cells associated with CHPF gene. **(J)** Correlation of CHPF methylation with immune cells.

In TCGA BRCA, we first analyzed the extent to which methylation occurred at different loci in the CHPF gene (**Figure 2A**). A subsequent correlation analysis revealed that CHPF transcript expression was negatively correlated with cg03176520 site methylation (**Figure 2B**). KM survival analysis showed that higher CHPF cg03176520 site methylation was correlated with better overall survival (OS) and progression-free survival (PFS) (**Figures 2C, D**) In addition, Breast cancer patients with both cg03176520 site hypermethylation and low CHPF gene expression had significantly increased overall survival contrasted to hypermethylation combined with high CHPF gene expression group (**Figure 2E**). We also analyzed immune cell infiltration in tumor microenvironment. We can see that the degree of immune cell infiltration in the breast cancer tumor microenvironment correlates with prognosis(P=0.011) (**Figure 2F**). The results of immune cell content analysis based on high and low CHPF gene expression groups showed that a total of 10 immune cells differed between the two groups (P<0.05) (**Figure 2G**). The correlation test further analyzed the correlation between immune cells and the CHPF gene, and the results showed that a total of 12 immune cells were correlated with the target gene (P<0.05). In this time, a total of 10 differentially expressed immune cells were obtained after taking the intersection of immune cell differential analysis and correlation analysis results (**Figure 2H**). KM survival analysis of these 10 differential immune cells showed among them B cells memory, B cells naive, T cells CD4 memory resting, Macrophages M0, Macrophages M1 were statistically significant in relation to survival, where higher Macrophages M0, Macrophages M1, B cells memory were associated with poor prognosis (**Figure 2I**). Apart from that, the results from the GSCA database analysis showed that CHPF DNA methylation was significantly negatively correlated with the infiltration levels of B, CD8+ T/CD4+ T cells, cytotoxic, and Exhausted (**Figure 2J**).

### CHPF Gene Expression and CHPF DNA Methylation Correlate With Clinicopathological Parameters

**Figure 3:**
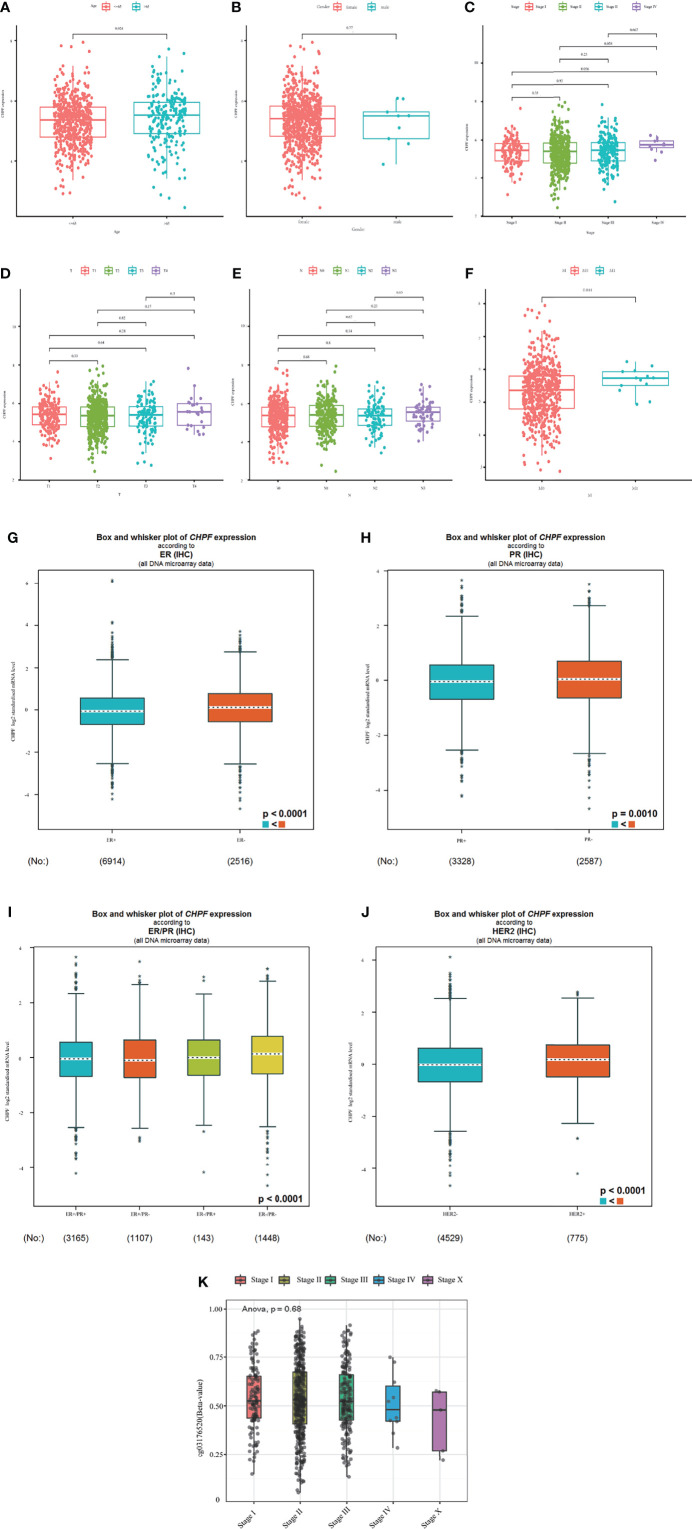
Expression levels of CHPF genes and CHPF DNA methylation in each clinical subgroup. **(A)** CHPF expression levels in age subgroups. **(B)** CHPF expression levels in M-staging subgroups, **(C)** CHPF expression levels in gender subgroups. **(D)** CHPF expression levels in T-stage subgroups. **(E)** CHPF expression levels in N-stage subgroups. **(F)** CHPF expression levels in tumor staging subgroups. **(G)** bc-GenExMiner analyzed the expression of CHPF genes under different ER states. **(H)** bc-GenExMiner analyzed the expression of CHPF genes under different PR states. **(I)** bc-GenExMiner analyzed the expression of CHPF genes under different ER/PR combinations. **(J)** bc-GenExMiner analyzed the expression of CHPF gene in HER2 subgroup. **(K)** Degree of CHPF methylation in tumor staging subgroups. *P < 0.05, **P < 0.01, ***P < 0.001.

We then analyzed the relationship between clinicopathological parameters and CHPF gene expression and methylation in BRCA patients. The results showed that CHPF expression was upregulated in patients over 35 years of age compared with patients <= 35 years of age (**Figure 3A**), and the level of CHPF expression was significantly higher in patients in stage M1 compared with the M0 group (**Figure 3B**). Although there were no significant differences in tumor stage, T-stage, and N-stage subgroups, CHPF expression appeared to be increased in stages IV, T4, and N3 compared to other classifications in the same group (**Figures 3C–F**). Using the online database bc-GenExMiner (http://bcgenex.ico.unicancer.fr), we investigated the relationship between CHPF expression and ER, PR, HER2, and from the results, we can conclude that CHPF is significantly predominantly present in ER-, PR-, ER/PR-, HER2+ groups, respectively (**Figures 3G–J**). And in clinical practice, these groups tend to have a poor prognosis. Meanwhile, the relationship between CHPF cg03176520 motif methylation and breast cancer tumor stage was analyzed online at smartapp (http://www.bioinfo-zs.com/smartapp/), and although not supported by positive results, we could still see that the CHPF cg03176520 motif was less methylated in samples with advanced IV samples were less methylated than other stages (**Figure 3K**).

### Correlation of CHPF Transcriptional Expression/DNA Methylation With Immune Markers

**Figure 4:**
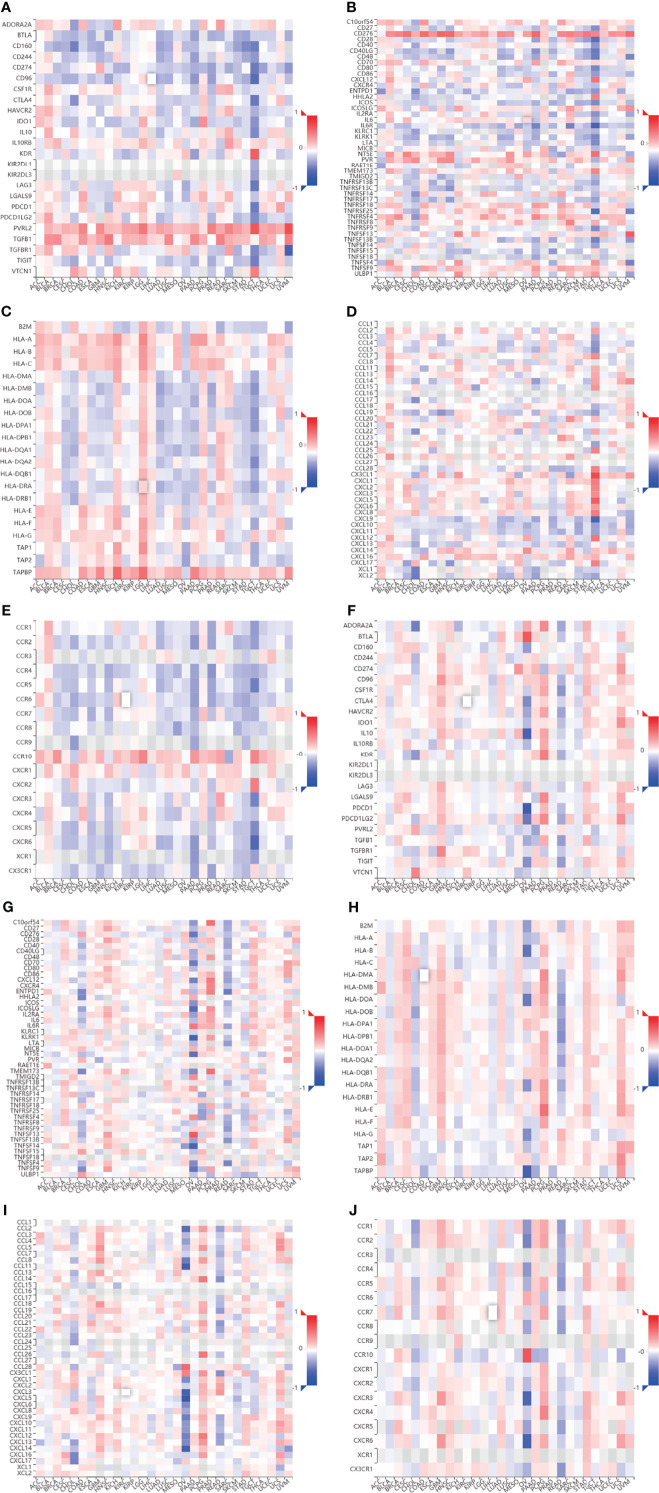
Correlation of CHPF transcriptional expression/DNA methylation with immune markers **(A)** Spearman correlations between expression of CHPF and immunoinhibitors. **(B)** Spearman correlations between expression of CHPF and immunostimulators. **(C)** Spearman correlations between expression of CHPF and MHCs. **(D)** Spearman correlations between expressions of CHPF and chemokine. **(E)** Spearman correlations between methylation of CHPF and receptor. **(F)**Spearman correlations between methylation of CHPF and immunoinhibitors. **(G)** Spearman correlations between methylation of CHPF and immunostimulators. **(H)** Spearman correlations between Methylation of CHPF and MHCs. **(I)** Spearman correlations between Methylation of CHPF and chemokine. **(J)** Spearman correlations between Methylation of CHPF and receptor.

We evaluated the relationship between CHPF transcript expression/DNA methylation and immune markers utilizing the TISIDB online database (**Figures 4A–J**). CHPF transcript expression is weakly correlated with immunomodulators such as BTLA, CD160, CD274, CD96, IL10RB, KDR, LGALS9, PVRL2, VSIR, CD40, CD40LG, CD70, IL6R, KLRK1, MICB, NT5E, PVR, TMEM173, TNFRSF14, TNFRSF18, TNFRSF25, TNFSF9, TNFSF13, TNFSF15, ULBP1 (1 < |R| < 3) and strongly correlated with CD276, TNFRSF4, TGFB1 (|R| > 3). While CHPF DNA methylation was only positive and unrelated to immunomodulators, there was no negative correlation (**Table 1**). CHPF transcript expression was weakly and positively correlated with the major histocompatibility complex (MHC)-associated molecules HLA-A, HLA-B, HLA-C, HLA-DPB1, HLA-DQB1, HLA-DRB1, HLA-E, HLA-F, HLA-G, TAPBP. Except for TAPBP, CHPF DNA methylation was significantly and negatively correlated with all MCH-associated molecules, especially with HLA-B, HLA-DMA, HLA-DMB, HLA-DOA, HLA-DOB, HLA-DPA1, HLA-DPB1, HLA-DQA1, HLA-DQB1, HLA-DRA, HLA-DRB1, HLA-E, HLA-F, TAP2 strongly correlated (**Table 2**). In terms of chemokines and receptors, CHPF transcript expression was weakly orthogonal to CCL7, CCL11, CX3CL1, CXCL16, strongly orthogonal to CCR10, and weakly negatively correlated with CXCL9, XCL1, CCR2CCR4, CCR5, CCR6, CCR7, CCR8. And CHPF DNA methylation was markedly positively correlated with most chemokines and receptors (**Table 3**). Interestingly, we discovered that immune-related molecules that were significantly associated with both CHPF transcriptional expression as well as CHPF DNA methylation always showed opposite trends.

**Table 1:** Correlation analysis between CHPF expression/DNA methylation and immunomodulators.

Immunomodulators	NEFM expression TISIDBrho, n=1100	p	NEFM DNA methylation TISIDBrho, n=1100	p
ADORA2A	0.045	0.138	0.103	**0.0038**
BTLA	-0.17	**1.34e^-08^**	0.147	**3.76e^−05^**
CD160	− 0.282	**1.71e^-21^**	0.103	**0.00372**
CD244	-0.026	0.391	0.132	**0.00022**
CD274(PD-L1)	− 0.205	**6.82e^-12^**	0.023	0.521
CD96	-0.153	**3.35e^-07^**	0.142	** 6.71e^-05^**
CSF1R	0.093	**0.00207**	0.116	**0.00109**
CTLA4	-0.009	0.76	0.096	**0.00719**
HAVCR2	0.071	**0.0181**	0.035	0.322
IDO1	− 0.039	0.199	0.099	** 0.00541**
IL10	0.011	0.724	0.045	0.211
IL10RB	0.234	**4.3e^-15^**	0.037	0.298
KDR(VEGFR)	-0.104	**0.000564**	− 0.001	0.974
LAG3	0.051	0.0925	0.075	**0.0358**
LGALS9	0.123	**4.19e^-05^**	0.143	**5.78e^-05^**
PDCD1	0.047	0.123	0.184	**2.31e^-07^**
PDCD1LG2	-0.036	0.227	0.038	0.291
PVRL2(NECTIN2)	0.171	**1.12 e^−08^**	0.053	0.137
TGFB1	0.418	** < 2.2e^−16^**	0.171	**1.51e^-06^**
TGFBR1	-0.003	0.925	-0.057	0.112
TIGIT	− 0.08	**0.00808**	0.111	**0.00188**
VTCN1	-0.014	0.635	0.075	**0.0347**
C10orf54(VSIR, VISTA)	0.175	**5.73e^−09^**	0.224	** 2.29e^−10^**
CD27(TNFRSF7)	-0.007	0.824	0.179	**4.71e^−07^**
CD276	0.465	** < 2.2e^−16^**	− 0.042	0.237
CD28	-0.071	**0.0185**	0.097	**0.00682**
CD40	0.115	**0.000132**	0.146	** 3.99e^-05^**
CD40LG	-0.14	**3.34e^-06^**	0.185	** 1.81e^−07^**
CD48	-0.069	**0.0212**	0.16	**6.88e^−06^**
CD70	0.208	**3.7e^-12^**	0.081	**0.0225**
CD80	− 0.026	0.388	− 0.039	0.27
CD86	0.02	0.514	0.031	0.383
CXCL12	0.056	0.063	0.08	**0.0256**
CXCR4	0.031	0.312	0.051	0.154
ENTPD1(CD39)	-0.099	**0.00104**	0.016	0.65
ICOS	− 0.085	**0.00472**	0.082	** 0.0211**
ICOSLG	0.095	**0.00168**	0.115	**0.0012**
IL2RA	− 0.006	0.847	0.069	0.0533
IL6	-0.011	0.721	0.092	**0.00984**
IL6R	− 0.205	**6.88e^-12^**	0.085	** 0.0176**
KLRC1	-0.092	**0.00231**	0.076	**0.0341**
KLRK1	-0.109	**0.00028**	0.139	9.22e^-05^
LTA	− 0.032	0.296	0.12	**0.000787**
MICB	-0.135	**6.89e^-06^**	0.034	0.337
NT5E(CD73)	0.108	**0.00034**	-0.017	0.639
PVR	0.154	**2.85e^−07^**	− 0.067	0.0599
RAET1E	0.043	0.154	0.012	0.728
TMEM173(STING)	0.178	**3.07e^-09^**	0.144	**4.99e^−05^**
TNFRSF13B	-0.007	0.819	0.218	**7.59e^−10^**
TNFRSF13C	-0.097	**0.0013**	0.153	** 1.65e^−05^**
TNFRSF14	0.199	**3.11e^-11^**	0.162	**4.93e^-06^**
TNFRSF17	-0.06	**0.0468**	0.105	** 0.00325**
TNFRSF18	0.202	**1.66e^-11^**	0.104	**0.0037**
TNFRSF25	0.14	**3.01e^-06^**	0.211	**2.53e^-09^**
TNFRSF4	0.42	**< 2.2e^−16^**	− 0.188	**1.28e^-07^**
TNFRSF8	0.084	**0.00554**	0.204	**8.62e^-09^**
TNFRSF9	-0.073	**0.0154**	0.04	0.264
TNFSF13	0.115	**0.000127**	0.007	0.854
TNFSF13B	− 0.049	0.101	0.012	0.735
TNFSF14	-0.058	0.0539	0.172	** 1.23e^−06^**
TNFSF15	-0.165	3.6e^-08^	0.035	0.327
TNFSF4	0.107	4e^−04^	− 0.075	**0.0346**
TNFSF9	0.275	**2.25e^-20^**	0.07	0.0513
ULBP1 (NKG2D)	0.127	**2.4e^-05^**	− 0.042	0.237

Significant P value < 0.05 is in bold.

**Table 2:** Correlation analysis between CHPF expression/DNA methylation and MCH-associated molecules.

MHC molecules	NEFM expression	*p*	NEFM DNA methylation	*p*
	TISIDB rho,		TISIDB rho,	
	n = 1100		n = 1100	
B2M	0.0570	0.0574	-0.2870	**<0.0001**
HLA-A	0.2370	**<0.0001**	-0.3000	**<0.0001**
HLA-B	0.1890	**<0.0001**	-0.3150	**<0.0001**
HLA-C	0.2630	**<0.0001**	-0.2120	**0.0003**
HLA-DMA	0.0920	**0.0022**	-0.4050	**<0.0001**
HLA-DMB	0.0080	0.7990	-0.4140	**<0.0001**
HLA-DOA	-0.0150	0.6260	-0.4580	**<0.0001**
HLA-DOB	-0.0220	0.4600	-0.5030	**<0.0001**
HLA-DPA1	-0.0040	0.9050	-0.4070	**<0.0001**
HLA-DPB1	0.1220	**0.0332**	-0.4350	**<0.0001**
HLA-DQA1	0.0080	0.8030	-0.3980	**<0.0001**
HLA-DQA2	-0.0090	0.7640	-0.2940	**<0.0001**
HLA-DQB1	0.1190	**0.0001**	-0.3520	**<0.0001**
HLA-DRA	-0.0040	0.8940	-0.4340	**<0.0001**
HLA-DRB1	0.1340	**<0.0001**	-0.3900	**<0.0001**
HLA-E	0.1590	**<0.0001**	-0.4840	**<0.0001**
HLA-F	0.209	**<0.0001**	-0.3770	**<0.0001**
HLA-G	0.2020	**<0.0001**	-0.2310	**<0.0001**
TAP1	0.073	**0.0153**	-0.2670	**<0.0001**
TAP2	0.042	0.1660	-0.3810	**<0.0001**
TAPBP	0.2160	**<0.0001**	-0.0570	0.1090

Significant P value < 0.05 is in bold.

**Table 3:** Correlation analysis between CHPF expression/DNA methylation and chemokines, receptors.

Chemokine	CHPF expression	*p*	CHPF DNA methylation	*p*
	TISIDBrho, n = 1100		TISIDBrho, n = 1100	
CCL2	0.025	0.417	0.09	**0.0118**
CCL3	0.089	**0.003**	0.049	0.172
CCL4	-0.001	0.964	0.074	**0.0394**
CCL5	-0.012	0.689	0.163	**<0.0001**
CCL7	0.11	**0.0003**	-0.004	0.912
CCL8	-0.003	0.911	0.02	0.584
CCL11	0.124	**<0.0001**	0.022	0.531
CCL13	-0.019	0.53	0.088	**0.0133**
CCL14	-0.035	0.252	0.184	**<0.0001**
CCL17	0.048	0.109	0.193	**<0.0001**
CCL18	0.01	0.742	0.081	**0.0236**
CCL19	-0.081	**0.0076**	0.224	**<0.0001**
CCL20	0.056	0.0628	0.034	0.343
CCL21	0.005	0.86	0.199	**<0.0001**
CCL22	0.019	0.535	0.119	**0.0009**
CCL28	-0.013	0.664	0.073	**0.0416**
CX3CL1	0.104	**0.0005**	0.14	**0.0001**
CXCL1	0.018	0.546	0.135	**0.0002**
CXCL2	0.012	0.681	0.132	**0.0002**
CXCL3	0.035	0.251	0.067	0.0615
CXCL5	0.011	0.715	0.073	**0.0405**
CXCL6	-0.038	0.21	0.099	**0.0056**
CXCL8	0.097	**0.0013**	-0.028	0.437
CXCL9	-0.109	**0.0003**	0.097	**0.0064**
CXCL10	-0.056	0.0622	0.034	0.343
CXCL11	-0.036	0.227	0.04	0.26
CXCL12	0.056	0.063	0.08	**0.0256**
CXCL13	-0.095	**0.0017**	0.092	**0.0097**
CXCL14	-0.006	0.844	0.053	0.136
CXCL16	0.162	**<0.0001**	0.066	0.0654
CXCL17	0.168	**0.0246**	0.052	0.145
XCL1	-0.101	**0.0008**	0.119	**0.0008**
XCL2	-0.049	0.102	0.122	**0.0006**
CCR1	-0.024	0.428	0.013	0.717
CCR2	-0.157	**<0.0001**	0.122	**0.0006**
CCR4	-0.167	**<0.0001**	0.114	**0.0014**
CCR5	-0.106	**0.0004**	0.119	**0.0008**
CCR6	-0.189	**<0.0001**	0.136	**0.0001**
CCR7	-0.117	**0.0001**	0.218	**<0.0001**
CCR8	-0.14	**<0.0001**	-0.001	0.986
CCR10	0.394	**<0.0001**	0.187	**<0.0001**
CX3CR1	-0.048	0.112	0.035	0.329
CXCR1	0.023	0.437	0.076	**0.0324**
CXCR2	-0.046	0.131	0.033	0.356
CXCR3	0.021	0.48	0.179	**<0.0001**
CXCR4	0.031	0.312	0.051	0.154
CXCR5	-0.047	0.12	0.212	**<0.0001**
CXCR6	-0.089	0.0031	0.099	**0.0057**

Significant P value < 0.05 is in bold.

### CHPF Promotes Breast Cancer Cell Proliferation, Migration, and Invasion *In Vitro*

**Figure 5:**
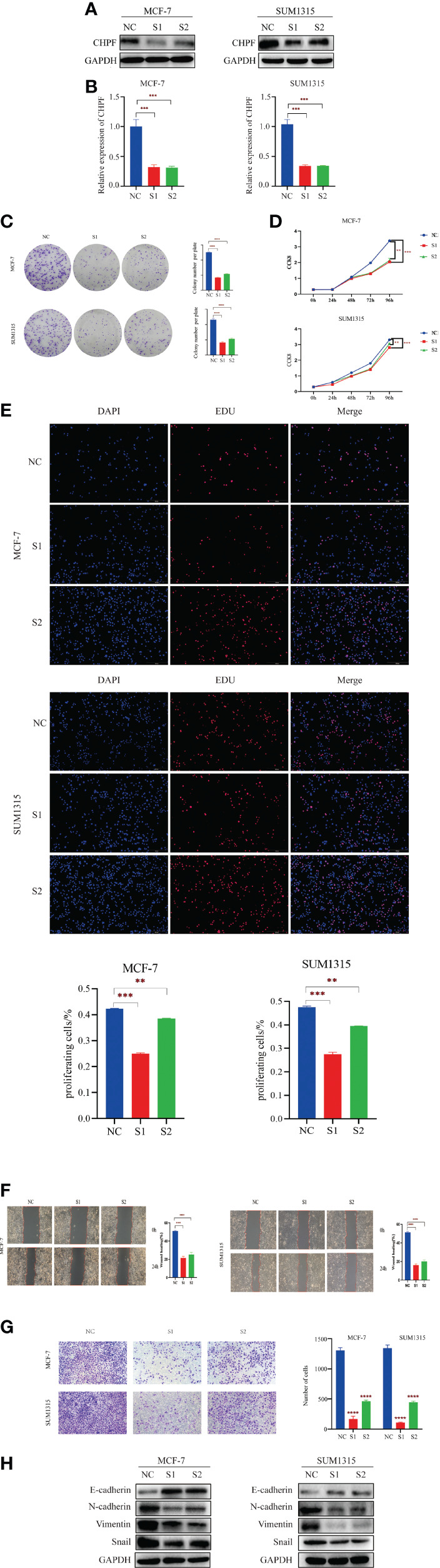
CHPF promotes proliferation, migration, and invasion of breast cancer cells. **(A)** Western Blotting for Validating the Knockdown Effect of CHPF Gene in Breast Cancer Cell Lines MCF-7 and SUM1315A. **(B)** Real-time quantitative PCR confirms changes in mRNA levels after knockdown of the CHPF gene in MCF-7 and SUM1315. **(C-E)** Clone formation experiments, CCK8, and EDU assay all showed that knockdown of the CHPF gene significantly inhibited the proliferation of breast cancer cells. **(F, G)** Wound healing, and invasion assays were performed to identify metastasis ability after CHPF knockdown in MCF-7 and SUM1315 cells. **(H)** Changes in the expression of the EMT biomarkers E-cadherin, N-cadherin, Snail, and Vimentin after CHPF knockdown were detected by western blot. All experiments were repeated three times. The data are shown as mean ± S.D. *P < 0.05, **P < 0.01, ***P < 0.001, and ****P < 0.0001.

Since the expression of CHPF was higher in MCF-7 cells and SUM1315 cells than in normal mammary cells MCF-10A in the cell line validation, these two cell lines were selected for interference and transiently transfected with siRNA targeting CHPF (S1 and S2) or siCtrl (NC) in MCF-7 and SUM1315 cells, respectively. Both western blot and RT-qPCR results showed that CHPF was significantly reduced in CHPF siRNA-transfected cells compared to control cells (**Figures 5A, B**) The results of the clone formation assay showed that the number of colony formation was dramatically reduced in the knockdown CHPF group compared to the control group (**Figure 5C**). The proliferation of MCF-7 and SUM1315 cells was markedly decreased after down-regulation of CHPF expression in the CCK8 value-added assay (**Figure 5D**). In addition, EDU incorporation analysis also showed that the proportion of EDU-positive MCF-7 and SUM1315 cells was significantly reduced in the CHPF-interfered group compared with the corresponding control cells (**Figure 5E**). The results of wound healing and invasion assays showed that interference with CHPF reduced the migratory capacity and invasive ability of MCF-7 and SUM1315 cells (**Figures 5F, G**). Furthermore, the protein levels of EMT-related genes N-cadherin, Snail, and Vimentin were down-regulated in CHPF knockdown MCF-7 and SUM1315 cells, while the protein levels of E-cadherin were up-regulated (**Figure 5H**).

### CHPF Can Alter the Expression of Genes Related to ECM-Receptor Interactions and PI3K-AKT Pathways

**Figure 6:**
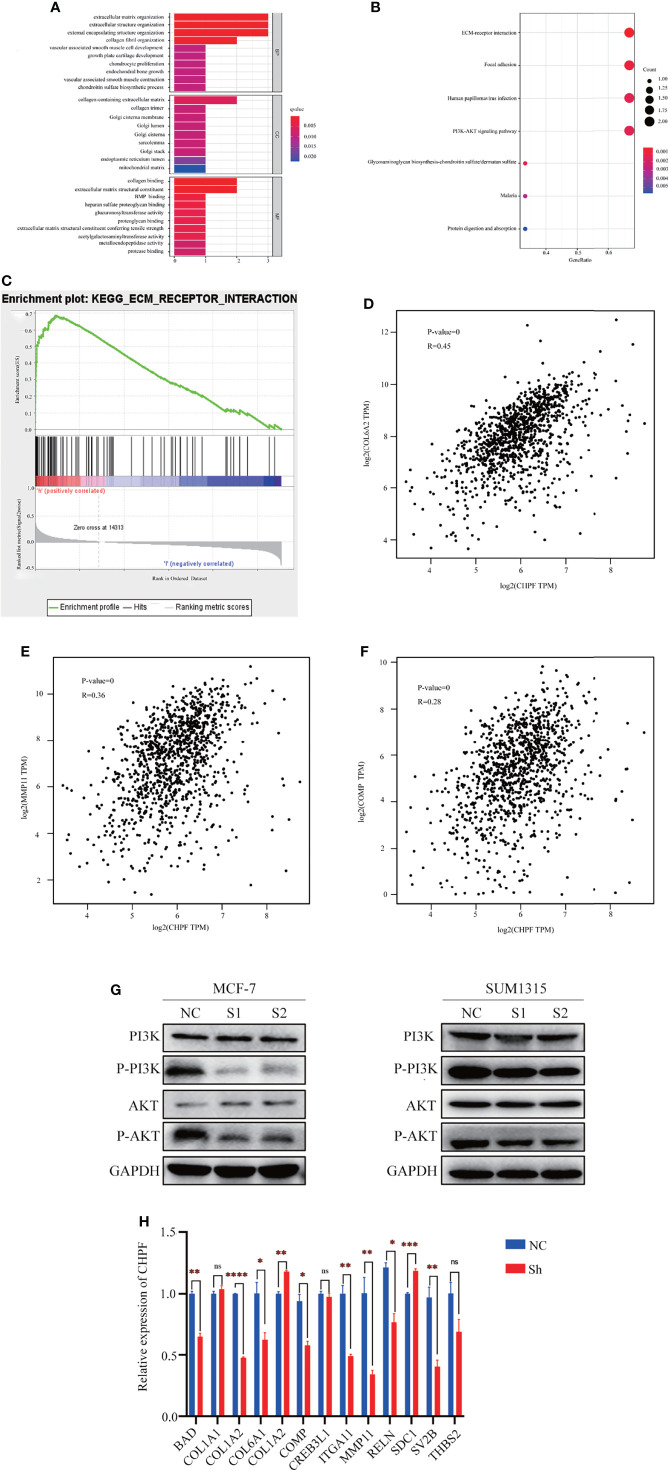
The results of enrichment analysis of CHPF-related genes GO, KEGG pathway. **(A)** Bar plot of GO enrichment analysis results. **(B)** Bubble plot of KEGG enrichment analysis results. **(C)** GSEA enrichment analysis results. **(D)** Correlation analysis of CHPF gene and COL6A2 gene. **(E)** Correlation analysis of CHPF gene and MMP11 gene. **(F)** Correlation analysis of CHPF gene and COMP gene. **(G)** Changes in protein levels of critical genes in the PI3K-AKT signaling pathway. **(H)** Results of quantitative real-time PCR of genes differentially expressed and involved in ECM-receptor interactions and PI3K-AKT pathway. *P < 0.05, **P < 0.01, ***P < 0.001, ****P < 0.0001. ns is the abbreviated form of non-significance.

To further understand the molecular mechanism of CHPF-induced BRCA metastasis, we performed bioinformatics analysis using TCGA-BRCA data. The samples were divided into two groups of high and low CHPF gene expression, and all genes in the two groups were analyzed for differential expression, with |logFC|>1 and adjusted P value <0.05 as the screening conditions, and a total of three differentially expressed genes were screened out, namely MMP11, COMP, and COL6A2. GO enrichment analysis revealed that the differential genes were mostly related to extracellular matrix-associated terms which are often associated with tumor aggressiveness (**Figure 6A**), while KEGG pathway enrichment analysis showed that differential genes were mainly enriched in ECM-receptor interaction and PI3K-AKT pathway (**Figure 6B**). The ECM-receptor interaction and PI3K-AKT pathways are cross-linked with each other and consist of many genes involved in cell motility and cancer metastasis, which is consistent with the metastasis-promoting role of CHPF genes. GSEA enrichment analysis was performed on the most significantly enriched ECM-receptor interaction pathway in KEGG, and the results showed a positive correlation between this pathway and CHPF gene expression. We used GEPIA to verify the correlation between the above three genes and the CHPF gene, and the results showed that all the above genes were significantly associated with the CHPF gene (**Figures 6C–F**).

In addition, western blotting was performed to verify the key genes of the PI3K-AKT signaling pathway. According to the results, it can be seen that the levels of P-PI3K and P-AKT in the CHPF knockdown group were lower than those in the control group (**Figure 6G**). Subsequently, we selected genes differentially expressed in the two groups and involved in the ECM-receptor interaction and PI3K-AKT pathways for quantitative real-time PCR validation (fold change > 1.5) (**Figure 6H**). We analyzed the changes in mRNA levels of a total of 13 genes this time, 10 of which were altered with the CHPF gene alterations. Among them, only COL6A2, and SDC1 were significantly increased in response to CHPF knockdown. In contrast, COL6A2 was indeed positively correlated with CHPF in the GEPIA database, which is contrary to our present findings.

## Discussion

Little has been reported about CHPF as a novel tumor-associated gene. At present, only a few publications focus on the role of CHPF in non-small cell adenocarcinoma, hepatocellular carcinoma, and breast cancer.

DNA methylation, an epigenetic modification (37–40). It plays a crucial role in normal human growth and development and cell biology (41, 42). Emerging evidence suggests that tumors often hijack various epigenetic mechanisms to evade immune restriction (38, 43). There are precise patterns of DNA methylation regulation in healthy human tissues, and changes in them can be detected in cancer development and progression. Previous studies have reported that hypomethylation of oncogenes is one of the hallmarks of almost all types of cancers, including breast cancer (44). Currently, there are no relevant studies on the methylation of this gene and immune infiltration.

The tumor microenvironment (TME) includes tumor cells, various immune cells as well as endothelial cells, and fibroblasts (45, 46). Previous studies have reported that TME components, particularly immune cells, influence tumor development and the body’s response to immune checkpoint blockade (ICB) (45).

In the current study, CHPF transcript expression was negatively correlated with DNA methylation in breast cancer, and CHPF transcript expression was associated with poorer prognosis while methylation of the CHPF DNA cg03176520 locus was associated with better survival. Immune cell differential analysis and correlation analysis showed that CHPF transcript expression was associated with 10 immune cells, including macrophages, CD4+ T cells, CD8+ T cells, and B cell infiltration. CHPF DNA methylation was significantly and negatively associated with B, CD8+ T/CD4+ T cells, cytotoxic, and Exhausted. In addition, CHPF transcript expression and DNA methylation correlated with various immunomodulators and most chemokines and receptors listed in TISIDB. Our study provides new research direction for the role of CHPF in breast cancer.

Our research also proved a significant increase in CHPF expression in breast cancer tissues. *In vitro*, CHPF promoted proliferation, migration, and invasion of breast cancer cells. The EMT-related genes associated with migration and invasion were also positive in this experiment. In addition, possible mechanisms were further investigated by bioinformatics analysis. We divided TCGA-BRCA samples into two groups of high and low expression according to the median expression of CHPF, and performed differential expression analysis of genes in both groups, and a total of three significantly different genes were obtained this time, namely MMP11, COMP, and COL6A2. Based on these three differential genes and the target gene CHPF, GO, KEGG, GSEA enrichment analysis was subsequently performed, and the results showed significant enrichment in ECM-receptor interactions and PI3K-AKT signaling pathways. After the knockdown of CHPF, we confirmed the changes in expression of key genes in the PI3K-AKT pathway, especially P-PI3K, P-AKT by western blotting, and the changes in differential genes in ECM-receptor interactions and PI3K-AKT pathway by RT-qPCR. The results showed that BAD, COL1A2, COL6A1, COL6A2, COMP, ITGA11, MMP11, RELN, SDC1, and SV2B were significantly differentially expressed. Moreover, among them, COL6A2, and SDC1 were significantly increased with the knockdown of the CHPF gene. In summary, we speculate that the CHPF gene may function with the above three genes, especially the more significantly altered MMP11, and subsequently promote breast cancer metastasis through the PI3K/AKT pathway. Of course, this requires further experimental validation.

There are also shortcomings in the present study, and the following questions still need to be addressed: (1) How the CHPF gene plays a role in promoting the proliferation and migration invasion of breast cancer cells with the help of the PI3K-AKT pathway, and whether it must act through the relevant genes we have validated need to be further investigated. (2) There is a lack of support from animal experiments. In addition, in this study, no experiments such as cell cycle were performed to verify that CHPF promotes cell proliferation (3) The CHPF gene has been linked to immunity in both transcriptional expression and DNA methylation, and the next step is to find the most relevant immune markers for the target gene and to investigate whether CHPF affects certain immunotherapy targets. (4) In addition, we have only explored the relationship between CHPF and immunity initially by bioinformatics methods. Relevant experimental evidence, such as the use of immunohistochemistry or PCR to verify the association between CHPF and immune-inflammatory indicators, is still lacking. The above shortcomings will require more time and effort to explore further in the future.

## Conclusion

In conclusion, our study demonstrated that CHPF, a novel tumor-promoting gene in BRCA, can promote cell migration and invasion through the ECM and PI3K/AKT signaling pathways, ultimately altering the survival of BRCA patients. Our findings highlight the critical role of CHPF in BRCA metastasis and its potential prognostic and therapeutic value.

## Data Availability Statement

The original contributions presented in the study are included in the article/**Supplementary Material**. Further inquiries can be directed to the corresponding authors.

## Author Contributions

W-WL: drafting paper design, article ideas, data collection, and writing article. BL: revision of thesis. S-QD: data collection and collection of clinical specimens. S-QH: data collection and collection of clinical specimens. Y-YL: data collection and collection of clinical specimens. S-YW: data collection and collection of clinical specimens. J-YM: data collection and collection of clinical specimens. J-XZ: revision of thesis and final version of paper approved for publication. ZL: revision of thesis and final version of paper approved for publication. All authors contributed to the article and approved the submitted version.

## Funding

Jiangsu Province Traditional Chinese Medicine Science and Technology Development Plan, MS2021101, Chinese Young Breast Experts Research project, CYBER-2021-010, The Key Research and Development Plan of Xuzhou KC21218, and National Natural Science Foundation of China 81602321.

## Conflict of Interest

The authors declare that the research was conducted in the absence of any commercial or financial relationships that could be construed as a potential conflict of interest.

## Publisher’s Note

All claims expressed in this article are solely those of the authors and do not necessarily represent those of their affiliated organizations, or those of the publisher, the editors and the reviewers. Any product that may be evaluated in this article, or claim that may be made by its manufacturer, is not guaranteed or endorsed by the publisher.
