# Supplementary material for: Bioinformatics and Experimental Analysis of the Prognostic and Predictive Value of the CHPF Gene on Breast Cancer
Source: Front Oncol. 2022 Mar 15;12:856712. doi: 10.3389/fonc.2022.856712 (PMC8965246; doi:10.3389/fonc.2022.856712)
Supplement: Supplementary file 1 [file Table_1.docx]

**Table S1 The primer sequences**

| Gene | FORWARD | REVERSE |
| --- | --- | --- |
| COL1A1 | GAACAGGGCGACAGAGGCATAAAG | CAACAGGACCAGCATCACCAGTG |
| COL6A2 | CCAGGACACCATCAACCGCATC | CGGCTCCAAATTCACCCTTCTCTC |
| COMP | AGGACAGTGATGGCGATGGTATAGG | GAGCGTGACTTCAGCGTTCTCC |
| COL1A2 | TCCTTCTGGTCCTGTTGGTCCTG | AAGTCATAACCACCACCGCTTACAC |
| COL6A1 | GATAAACGGCACGAAGGGCTACC | GACGACGAAGTCCTTGGCAATCTC |
| THBS2 | GCCTGCTCCGTGGACATTGATG | TCCTCCTGGTCTGGGTTGAACAC |
| ITGA11 | CCATCTACATCTTCCACGGCTTCC | GGAGGCTGGCATTGATCTGAACC |
| BAD | CCAACCAGCAGCAGCCATCATG | GTCCACAAACTCGTCACTCATCCTC |
| CREB3L1 | AGGAGAGCCGTCGTAAGAAGAAGG | GGAGCCCAGCACCAGAACAAAG |
| SDC1 | GAGAGGGCTGCTGAGGATGGAG | GTTTGGTGGGCTTCTGGTAGGC |
| RELN | CACTCTGCCTCTCCCTCCTTATACC | CTCCGTTCACAGTCAGCCAGTTC |
| SV2B | AGCCTTCCTCTTCTGCCGACTC | AGCACAGACGATGACAAACACTCTC |
| MMP11 | GCCCTAAAGGTATGGAGCGATGTG | CAGTGGGTAGCGAAAGGTGTAGAAG |
| GAPDH | GGAGCGAGATCCCTCCAAAATATCCCTCCAAAAT | GGCTGTTGTCATACTTCTCATGG |
| CHPF | CCTGCTGCTACTGTATGAG | GCTCCTCTTCTTGTTCTGA |

**Table S2 The siRNA sequences**

| SiRNA | sense |
| --- | --- |
| SiLCHPF#1 | AGCTGGCCATGCTACTCTTTG |
| SiLCHPF#2 | CUGGCCAUGCUACUCUUUGTT |
| siCtrl | UUCUCCGAACGUGUCACGUTT |

**Table S3** **The shRNA target sequences**

| shRNA | sense |
| --- | --- |
| shCHPF#1 | GCTGTGGCCTCCACGTATTTA |
| shCHPF#2 | AGCTGGCCATGCTACTCTTTG |
| shCtrl | TTCTCCGAACGTGTCACGT |
